# EPA guidance on treatment of negative symptoms in schizophrenia

**DOI:** 10.1192/j.eurpsy.2021.13

**Published:** 2021-03-17

**Authors:** S. Galderisi, S. Kaiser, I. Bitter, M. Nordentoft, A. Mucci, M. Sabé, G. M. Giordano, M. Ø. Nielsen, L. B. Glenthøj, P. Pezzella, P. Falkai, S. Dollfus, W. Gaebel

**Affiliations:** 1 Department of Psychiatry, University of Campania Luigi Vanvitelli, Naples, Italy; 2 Division of Adult Psychiatry, Department of Psychiatry, Geneva University Hospitals, Geneva, Switzerland; 3 Department of Psychiatry and Psychotherapy, Semmelweis University, Budapest, Hungary; 4 Copenhagen Research Centre for Mental Health (CORE), Copenhagen University Hospital, Copenhagen, Denmark; 5 Department of Clinical Medicine, Faculty of Health and Medical Science, University of Copenhagen, Copenhagen, Denmark; 6 Centre for Clinical Intervention and Neuropsychiatric Schizophrenia Research, CINS, Glostrup, Denmark; 7 Center for Neuropsychiatric Schizophrenia Research, CNSR, Glostrup, Denmark; 8 Department of Psychiatry, University of Munich, Munich, Germany; 9 Service de Psychiatrie, CHU de Caen, 14000 Caen, France; 10 Normandie Univ, UNICAEN, ISTS EA 7466, GIP Cyceron, 14000 Caen, France; 11 Normandie Univ, UNICAEN, UFR de Médecine, 14000 Caen, France; 12 Department of Psychiatry and Psychotherapy, Medical Faculty, Heinrich-Heine University, Düsseldorf, Germany

**Keywords:** Negative symptoms, schizophrenia, treatment

## Abstract

Negative symptoms of schizophrenia remain a major therapeutic challenge. The progress in the conceptualization and assessment is not yet fully reflected by treatment research. Nevertheless, there is a growing evidence base regarding the effects of biological and psychosocial interventions on negative symptoms. The importance of the distinction between primary and secondary negative symptoms for treatment selection might seem evident, but the currently available evidence remains limited. Good clinical practice is recommended for the treatment of secondary negative symptoms. Antipsychotic treatment should be optimized to avoid secondary negative symptoms due to side effects and due to positive symptoms. For most available interventions, further evidence is needed to formulate sound recommendations for primary, persistent, or predominant negative symptoms.

However, based on currently available evidence recommendations for the treatment of undifferentiated negative symptoms (including both primary and secondary negative symptoms) are provided. Although it has proven difficult to formulate an evidence-based recommendation for the choice of an antipsychotic, a switch to a second-generation antipsychotic should be considered for patients who are treated with a first-generation antipsychotic. Antidepressant add-on to antipsychotic treatment is an option. Social skills training is recommended as well as cognitive remediation for patients who also show cognitive impairment. Exercise interventions also have shown promise. Finally, access to treatment and to psychosocial rehabilitation should be ensured for patients with negative symptoms. Overall, there is definitive progress in the field, but further research is clearly needed to develop specific treatments for negative symptoms.

## Introduction

Negative symptoms, including loss of motivation and reduction of emotional expression, represent a core aspect of schizophrenia [1–3]. They are associated with low remission rates, poor real-life functioning, and quality of life and place a substantial burden on patients, relatives, and health care systems. For this reason, negative symptoms have become a key target in the search for new therapeutic tools. However, so far, progress in the development of innovative treatments has been slow and negative symptoms often represent an unmet need in the care of subjects with schizophrenia [4–8]. Progress occurred in the assessment of negative symptoms, as outlined in the European Psychiatric Association (EPA) guidance on the assessment of negative symptoms [9], has not yet reached the same level of specificity in treatment research [4,10,11]. The large majority of studies does neither differentiate primary from secondary symptoms, nor does it control for the major sources of secondary negative symptoms. Some treatment studies define a minimum severity and duration of negative symptoms, but the criteria are very heterogeneous. Furthermore, the two- or five-factor model of negative symptoms has rarely been applied in treatment studies.

Fusar-Poli and colleagues have published a meta-analysis of placebo-controlled trials investigating a wide range of interventions that illustrates the challenges in research on treatment of negative symptoms [12]. They found a statistically significant effect for combination treatment, antidepressants, second-generation antipsychotics, and psychosocial treatments. Several problems preclude deriving concrete clinical recommendations from this meta-analysis, most of which are discussed in the article. First, it was not possible to differentiate between primary and secondary negative symptoms or to identify effects on prominent or predominant negative symptoms. Second, the trial duration was heterogeneous and overall trial duration was short. Third, the intervention categories were very heterogeneous. Fourth, the authors did not consider the observed effects to be clinically meaningful. However, given the heterogeneity of the included studies and some methodological issues [13,14], it seems very difficult to effectively determine such a threshold. Fifth, the most frequently used rating scales for the measurement of negative symptoms (Brief Psychiatric Rating Scale (BPRS), Positive and Negative Syndrome Scale (PANSS), and Scale for the Assessment of Negative Symptoms (SANS)) are associated with significantly different effect sizes in the respective studies [15] and there are too few data with the recently introduced Brief Negative Symptom Scale and Clinical Assessment Interview for Negative Symptoms to compare them with earlier efficacy data (effect sizes) based on the use of other scales.

In our review of the evidence, we focused on meta-analyses with more narrowly defined categories of interventions and patient populations, but it was rarely possible to address the challenges mentioned above in a satisfactory way. Therefore, the working group decided to organize the present treatment guidance by starting the review of the evidence for each intervention with general negative symptoms without differentiation. Specific recommendations are considered only where evidence on the treatment of specific forms of negative symptoms is available and the need for research is pointed out where this is not the case. Please note that we considered the potential risks associated with the different interventions for the formulation of the recommendations.

## Methodology

### Systematic literature search

The development of EPA guidance on the treatment of negative symptoms followed the standardized methods, according to the European Guidance Project of the EPA and to the Preferred Reporting Items for Systematic reviews and Meta-Analyses, as described in previous publications [16–20].

In brief, we performed a comprehensive literature search on treatment of the negative symptoms in subjects with schizophrenia. The search has been run in three electronic databases: Medline (PubMed), Scopus, and PsycINFO, with the aim to ensure that it was as comprehensive as possible (Table 1). The search was conducted on December 9, 2019 with no limitation regarding the starting date.

**Table 1.** Systematic search strategies.

Database	Search syntax	Number of retrieved documents	Date of search
Medline (PubMed)	(Schizophrenia AND “negative symptoms”) OR (Schizophrenia AND avolition) OR (Schizophrenia AND apathy) OR (Schizophrenia AND anhedonia) OR (Schizophrenia AND alogia) OR (Schizophrenia AND asociality) OR (Schizophrenia AND amotivation) OR (Schizophrenia AND “social withdrawal”) OR (Schizophrenia AND “blunted affect”) OR (Schizophrenia AND “affective flattening”) OR (Schizophrenia AND “persistent negative symptoms”) OR (Schizophrenia AND “predominant negative symptoms”) OR (Schizophrenia AND “prominent negative symptoms”) OR (Schizophrenia AND “primary negative symptoms”) OR (Schizophrenia AND “deficit schizophrenia”) OR (Schizophrenia AND “lack of motivation”)	6,438	December 9, 2019
	Filters: Languages, English; Species, Human		
	Search in [Title/Abstract]		
	No time limit			
Scopus	(Schizophrenia AND “negative symptoms”) OR (Schizophrenia AND avolition) OR (Schizophrenia AND apathy) OR (Schizophrenia AND anhedonia) OR (Schizophrenia AND alogia) OR (Schizophrenia AND asociality) OR (Schizophrenia AND amotivation) OR (Schizophrenia AND “social withdrawal”) OR (Schizophrenia AND “blunted affect”) OR (Schizophrenia AND “affective flattening”) OR (Schizophrenia AND “persistent negative symptoms”) OR (Schizophrenia AND “predominant negative symptoms”) OR (Schizophrenia AND “prominent negative symptoms”) OR (Schizophrenia AND “primary negative symptoms”) OR (Schizophrenia AND “deficit schizophrenia”) OR (Schizophrenia AND “lack of motivation”)	9,863	December 9, 2019
	Filters: Languages, English; Species, Human		
	Search in [Title/Abstract/Keywords]		
	No time limit		
PsychINFO	(Schizophrenia AND “negative symptoms”) OR (Schizophrenia AND avolition) OR (Schizophrenia AND apathy) OR (Schizophrenia AND anhedonia) OR (Schizophrenia AND alogia) OR (Schizophrenia AND asociality) OR (Schizophrenia AND amotivation) OR (Schizophrenia AND “social withdrawal”) OR (Schizophrenia AND “blunted affect”) OR (Schizophrenia AND “affective flattening”) OR (Schizophrenia AND “persistent negative symptoms”) OR (Schizophrenia AND “predominant negative symptoms”) OR (Schizophrenia AND “prominent negative symptoms”) OR (Schizophrenia AND “primary negative symptoms”) OR (Schizophrenia AND “deficit schizophrenia”) OR (Schizophrenia AND “lack of motivation”)	10,481	December 9, 2019
	Filters: Languages, English; Species, Human		
	Search in [Title/Abstract/Keywords]		
	No time limit		

**Figure 1.**
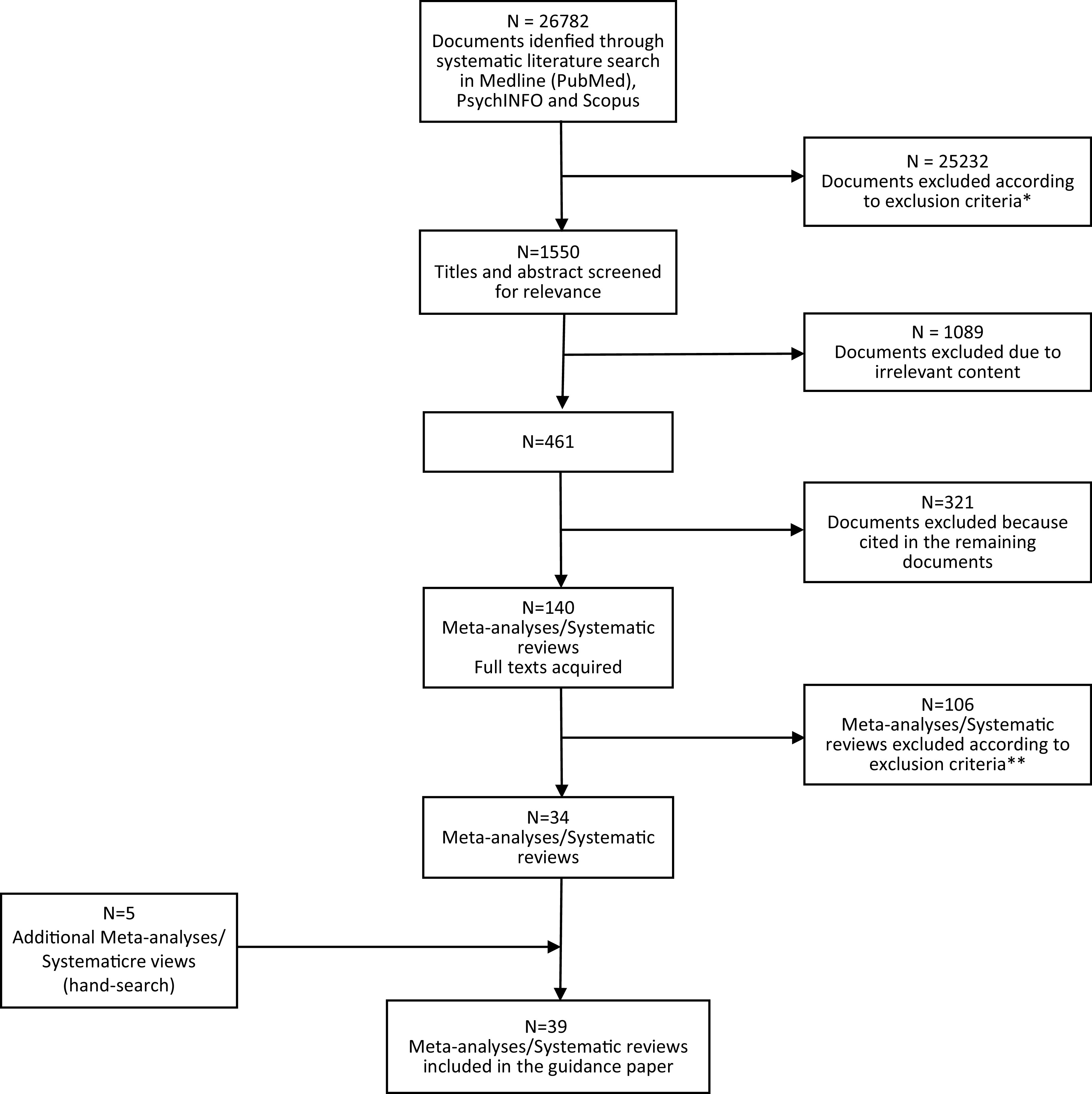
Preferred reporting items for systematic reviews and meta-analyses flowchart of studies retrieved in the systematic literature search. *11,905 duplicates; 1,826 studies other than meta-analysis, randomized controlled trial, review, cohort study, open study, descriptive study, expert opinion; 843 studies published in journal not indexed in Embase or Medline; 2,895 studies on pathophysiological mechanisms of negative symptoms; 5,813 articles not related to any topic; 1,792 articles related to the assessment of negative symptoms; 158 studies conducted in animals. **Outdated; concerns about quality of meta-analytic procedures or of the original studies; addressed population/intervention/outcome not usable for formulation of recommendations.

A database was created with studies selected according to predefined inclusion and exclusion criteria as follows (see Figure 1 for details on the selection process):

### Inclusion criteria

1. meta-analysis, randomized controlled trial (RCTs), review, cohort study, open study, descriptive study, and expert opinion concerning the treatment of negative symptoms in subjects with schizophrenia according to the search terms cited in Table 1;
2. studies published in English;
3. studies carried out in humans;
4. studies published in journals indexed in Embase or Medline.

### Exclusion criteria

1. duplicates, comments, editorials, case reports/case series, theses, proceedings, letters, short surveys, notes;
2. studies irrelevant for the topic, including studies relevant to the conceptualization and assessment of negative symptoms;
3. studies concerning exclusively pathophysiological mechanisms of negative symptoms (those reporting imaging or electrophysiological or other biomarker correlates of negative symptoms);
4. unavailable full-text;
5. studies that do not meet inclusion criteria.

The present recommendations for treatment were based on the most recent high-quality meta-analyses available for each treatment modality. An additional search for RCTs was conducted for the time period after the search period of the most recent meta-analysis using its search terms and reference lists were hand-searched to identify additional publications missed by the search strategy.

Meta-analytic studies were not used to formulate recommendations if the following criteria were fulfilled: (a) outdated, that is, replaced by a more recent meta-analysis on the treatment modality, (b) concerns about quality of meta-analytic procedures or of the original studies, and (c) addressed population/intervention/outcome not usable for formulation of recommendations. More than one meta-analysis could be included in the recommendations for the same treatment modality. Lower level evidence (i.e., non-randomized or uncontrolled trials, case reports) was not included in the formulation of the recommendations.

The final set of included studies was graded for the level of evidence by three authors (SK, IB, and MN) following Gaebel et al. [21] (Table 2). These three authors also developed an initial formulation of the recommendations based on the evidence level of the included studies (Table 3 for grading of recommendations). Then, the evidence grading and the recommendations were reviewed by all the other coauthors. Discrepancies in the ratings were resolved by discussion among all coauthors.

**Table 2.** Grading of evidence.

Grade	Features of quantitative studies	Features of reviews
I—Generalizable studies	Randomized controlled trials. Surveys sampling a large and representative group of persons from the general population or from a large range of service settings. Analytic procedures comprehensive and clear usually including multivariate analyses or statistical modeling. Results can be generalized to settings or stakeholder groups other than those reported in the study.	Systematic reviews or meta-analyses
II—Conceptual studies	Uncontrolled, blinded clinical trials. Surveys sampling a restricted group of persons or a limited number of service providers or settings. May be limited to one group about which little is known or a number of important subgroups. Analytic procedures comprehensive and clear. Results have limited generalizability.	Unsystematic reviews with a low degree of selection bias employing clearly defined search strategies
III—Descriptive studies	Open, uncontrolled clinical trials. Description of treatment as usual. Survey sampling not representative since it was selected from a single specialized setting or a small group of persons. Mainly records experiences and uses only a limited range of analytical procedures, like descriptive statistics. Results have limited generalizability.	Unsystematic reviews with a high degree of selection bias due to undefined or poorly defined search strategies
IV—Single case study	Case studies. Provides survey data on the views or experiences of a few individuals in a single setting. Can provide insight in unexplored contexts. Results cannot be generalized.	Editorials

Note. Modified from Gaebel et al. [21].

**Table 3.** Grading of recommendations.

Grade	Description
A	At least on study or review rated as I and directly applicable to the target population OR a body of evidence consisting principally of studies and/or reviews rated as I, directly applicable to the target population, and demonstrating overall consistency of results.
B	A body of evidence including studies and/or reviews rated as II, directly applicable to the target population, and demonstrating overall consistency of results OR extrapolated evidence from studies and/or reviews rated as I or II.
C	A body of evidence including studies and/or reviews rated as II-III, directly applicable to the target population, and demonstrating overall consistency of results OR extrapolated evidence from studies and/or reviews rated as II or III.
D	Level of evidence rated as III or IV OR extrapolated evidence from studies and/or reviews rated as III or IV OR expert consensus.

Note. Modified from Gaebel et al. [21].

## Treatment of Secondary Negative Symptoms

### General principles

It is commonly acknowledged that major sources of secondary negative symptoms should be assessed and treated in patients presenting with negative symptoms. Some tentative algorithms have been proposed [11,22]. However, it is important to note that treatment in the specific clinical situation of a patient presenting with negative symptoms, which are subsequently identified as being secondary, has not been evaluated in clinical trials. Therefore, treatment of depression, positive symptoms, and side effects should follow general principles for their management. All recommendations have to be extrapolated or based on expert consensus. Since secondary negative symptoms are a common clinical problem, further research should be a priority.

***Recommendation 1*** [11]

**Table:** 

Grade	Recommendation
B	Depression, positive symptoms, and side effects should be treated in patients presenting with negative symptoms. The treatment of these problems should follow available guidelines for their treatment as there is no evidence for a specific approach in patients presenting with negative symptoms.

The EPA guidance group on negative symptoms considers treatment of depression, positive symptoms, and extrapyramidal or sedating side effects a priority for patients presenting with negative symptoms. However, no evidence was found to recommend a specific approach in patients with negative symptoms. Thus, the EPA guidance group recommends the optimization of antipsychotic treatment as well as treatment of depressive episodes in accordance with available guidelines.

### Negative symptoms secondary to depression

There are no clinical trials that have specifically addressed the treatment of negative symptoms considered to be secondary to depression. Therefore, recommendations have to be extrapolated from evidence on the treatment of depression in patients with schizophrenia. It has been suggested that some antipsychotics have a comparatively favorable effect on depressive symptoms in patients with schizophrenia (quetiapine, amisulpride, aripiprazole, clozapine, and olanzapine) [23,24]. However, this evidence has to be regarded with caution as depressive symptoms were not the primary outcome and no recent meta-analysis is available.

Two meta-analyses reporting depressive symptoms as primary or secondary outcome concluded that add-on antidepressants maybe an effective treatment, in particular for patients with clinically significant depressive symptoms [25,26]. However, another recent meta-analysis including studies in which an antidepressant was added to an already ongoing treatment did not confirm these results [27]. The same meta-analysis found evidence for a beneficial effect of antidepressant add-on for negative symptoms independently of co-occurring depressive symptoms (see section “*Antidepressants*”).

An earlier meta-analysis suggested a positive effect of cognitive behavioral therapy (CBT) interventions on depressive symptoms in patients with schizophrenia [28]. However, the included studies did not target depressive symptoms as primary outcome and more recent meta-analyses did not present results for depressive symptoms. A recent systematic review concluded that CBT specifically tailored to depressive symptoms may be an effective treatment of comorbid depression in patients with schizophrenia [29].

***Recommendation 2a*** [23–24]**.**

**Table:** 

Grade	Recommendation
B	In a patient presenting with negative symptoms and comorbid depression, a switch to an antipsychotic with antidepressant properties should be considered.

***Recommendation 2b*** [25–27]**.**

**Table:** 

Grade	Recommendation
B	In a patient presenting with negative symptoms and comorbid depression, add-on antidepressant treatment should be considered.

***Recommendation 2c*** [26]

**Table:** 

Grade	Recommendation
B	If the trial with an add-on antidepressant is not associated with an improvement of negative symptoms and/or depression, the antidepressant should be discontinued to avoid polypharmacy.

***Recommendation 2d*** [28–29]**.**

**Table:** 

Grade	Recommendation
B	In a patient presenting with negative symptoms and comorbid depression, cognitive behavior therapy should be considered.

### Negative symptoms secondary to positive symptoms

There are no clinical studies that specifically address the treatment of negative symptoms considered to be secondary to positive symptoms. However, there is evidence for improvement of negative symptoms in antipsychotic trials including patients during an acute psychotic episode [13]. It has been suggested that improvement of negative symptoms in this context is in part secondary to improvement of positive symptoms [30]. Therefore, antipsychotic treatment should be optimized by following existing recommendations. Although recent meta-analyses showed somewhat inconsistent results [31–33], clozapine remains the main recommendation for treatment-resistant positive symptoms (see section “*Treatment-resistance and the role of clozapine in the treatment of negative symptoms*” below for further detail).

Similar to antipsychotic treatment, there are no clinical trials on CBT specifically addressing the reduction of negative symptoms considered to be secondary to positive symptoms. However, CBT interventions primarily targeting positive symptoms have shown a reduction in negative symptoms that may have been secondary to improvement of positive symptoms [28,34].

***Recommendation 3a*** [13,30]

**Table:** 

Grade	Recommendation
C	In a patient presenting with negative symptoms that are considered to be secondary to positive symptoms, antipsychotic treatment can be optimized by following existing recommendations regarding dose range and switching of medications.

***Recommendation 3b***

**Table:** 

Grade	Recommendation
B	In a patient presenting with negative symptoms that are considered to be secondary to treatment-resistant positive symptoms, a trial with clozapine should be considered.

***Recommendation 3c*** [28,34]

**Table:** 

Grade	Recommendation
C	In patients with negative symptoms considered to be secondary to positive symptoms, CBT can be considered.

### Negative symptoms secondary to side effects

There are no clinical studies that specifically address the treatment of negative symptoms considered to be secondary to side effects of medication. In this context, secondary negative symptoms can be caused by different types of side effects, namely extrapyramidal side effects, sedation, and antipsychotic-induced amotivation, although the latter is somewhat controversial. Overall, the evidence base for the treatment of these side effects is very limited, and therefore an extrapolation to the treatment of secondary negative symptoms is not feasible [35,36]. The following recommendation is therefore based on expert consensus.

***Recommendation 4*** [35,36]

**Table:** 

Grade	Recommendation
C	If a patient with negative symptoms shows extrapyramidal and/or sedative side effects, a reduction of the antipsychotic dose or a switch to an antipsychotic with lower risk for extrapyramidal and/or sedative side effects can be considered.

## Biological Treatments

### Antipsychotics

Antipsychotic treatment generally improves negative symptoms secondary to positive symptoms during acute phases of schizophrenia, however, primary, enduring negative symptoms such as those present in the deficit syndrome do not respond to the vast majority of available antipsychotics, or the response may not be clinically meaningful. The comparison of the efficacy of various treatments for negative symptoms in schizophrenia is challenging due to the large heterogeneity of the studies, for example, the majority of the studies did not separate primary and secondary negative symptoms and different rating scales were used. We summarize below the evidence available at the time of the writing of this guideline about the use of antipsychotics in the treatment of negative symptoms in schizophrenia (see eTable 1).

#### Antipsychotic vs placebo

Most clinical trials comparing antipsychotic vs placebo have measured global negative symptoms, without specifying primary, persistent, or predominant negative symptoms. The meta-analysis by Fusar-Poli and colleagues included 10 placebo-controlled studies with first-generation antipsychotics (FGA) and 38 studies with second-generation antipsychotics (SGA) [12]. Their results suggested a small improvement with SGA, but not with FGA. The challenges for the interpretation of these data have been outlined in the introduction above. These challenges also apply to some extent to a recent meta-analysis by Leucht and colleagues that included placebo-controlled acute treatment trials and found a small effect of antipsychotics compared to placebo on negative symptoms Standardized Mean Difference (SMD) 0.35, Confidence Interval (CI) 0.31–0.40) that was not specific to SGA [13].

These meta-analyses highlight the importance of the selection and definition of negative symptoms for future studies. In particular, as recommended by the European Medicines Agency (EMA) [37], it is important to exclude patients with secondary negative symptoms from such studies or, if such patients are included, control for secondary negative symptoms should be provided by measuring their sources (e.g., depression or extrapyramidal symptoms) as recommended by the Food and Drug Administration [38].

A recent meta-analysis by Krause and colleagues specifically addressed studies including patients with predominant or prominent negative symptoms [39]. Amisulpride (in low doses) is the only marketed antipsychotic drug that has evidence to be considered significantly better than placebo in the treatment of negative symptoms defined as predominant negative symptoms (SMD 0.47, CI 0.23–0.71) [39,40]. All available studies, except a negative one, were sponsored by the manufacturer. Overall, very few drugs have been tested against placebo for the treatment of predominant or prominent negative symptoms [39].

#### Comparison between antipsychotics

There are few (and mainly small) face-to-face studies comparing the efficacy of antipsychotics on negative symptoms. In an earlier meta-analysis, Leucht and colleagues reported that the second-generation antipsychotics amisulpride, clozapine, olanzapine, and risperidone were more efficacious than first-generation antipsychotics [23]. However, in addition to the limited number of trials available, the limitations discussed for the placebo-controlled trials mentioned above apply here as well.

Since few head-to-head trials are available, Huhn and colleagues used network meta-analysis to compare antipsychotics in acute treatment studies [41]. The authors of this meta-analysis “included RCTs in adults with acute symptoms of schizophrenia and related disorders…” and “…excluded studies in patients with treatment resistance, first episode, *predominant negative* or depressive symptoms, concomitant medical illnesses, and relapse preventions studies.” (italic added by the authors of this paper). They observed some gradual differences in the effects of antipsychotics on negative symptoms in the selected group of patients. However, these differences were quite similar to changes in overall and positive symptoms. These authors conclude that it is impossible to clarify in populations with positive symptoms whether differences in negative symptoms relate to primary or secondary negative symptoms. In addition, they note that many antipsychotics improved depressive symptoms, which renders the differentiation of negative symptom effects even more complicated.

To our knowledge and based on the recent meta-analysis by Krause et al. [39], there is only one sufficiently powered head-to-head study, which applied the criteria recommended by EMA [37]: it included patients with enduring predominant negative symptoms and it was controlled for sources of secondary negative symptoms (positive symptoms, depression, and extrapyramidal symptoms). This manufacturer-sponsored study found cariprazine—a dopamine D3 and D2 receptor partial agonist with preferential binding to D3 receptors—significantly better than risperidone in the treatment of predominant negative symptoms as measured by a negative factor of the PANSS and functioning as measured by the Personal and Social Performance Score, respectively [39,42].

#### Combination of antipsychotics

Guidelines recommend antipsychotic monotherapy for the treatment of schizophrenia, however polypharmacy is widely practiced worldwide. Based on the real-life efficacy of polypharmacy in the treatment of schizophrenia, the rigid recommendations for monotherapy have been challenged [43]. Many patients with negative symptoms are poor responders or even nonresponders to antipsychotic monotherapy and receive various combinations of antipsychotics, which are not supported by evidence.

A recent meta-analysis by Galling and colleagues suggests that combination of a D2 antagonist with a D2 partial agonist may have a beneficial effect for the treatment of negative symptoms, but several limitations preclude formulating a recommendation [44]. The meta-analysis does not allow any conclusion on primary, predominant, or even prominent negative symptoms. It is worth noticing that an important number of trials targeted adverse effects and not efficacy.

Finally, the largest included trial targeted efficacy on overall symptoms and did not show any improvement of negative symptoms with aripiprazole add-on to risperidone or quetiapine [45].

Therefore, the working group does not recommend any combination of antipsychotics for the treatment of negative symptoms at the present state of knowledge.

#### Recommendations concerning antipsychotic treatment for negative symptoms

Deriving recommendations for the treatment of negative symptoms with antipsychotics has proven to be very difficult due to the challenges outlined above. The most frequent situation encountered by clinicians is a patient treated with an antipsychotic that continues to have negative symptoms. The available evidence is not sufficient to give an evidence-based recommendation in this type of situation. Nevertheless, all members of the EPA guidance group on negative symptoms agreed that for patients treated with first-generation antipsychotics a switch to a second-generation should be considered. Although the level of evidence would be compatible with a grade D recommendation, all group members agreed to upgrade the recommendation to grade B based on their knowledge and experience.

***Recommendation 5*** [12,23]

**Table:** 

Grade	Recommendation
B	For patients with negative symptoms who are treated with a first-generation antipsychotic, a switch to a second-generation antipsychotic should be considered.

Please note that the working group could not recommend a specific second-generation antipsychotic. While there is certainly potential for amisulpride and cariprazine for the treatment of predominant and persistent negative symptoms, further research is necessary before a specific recommendation can be provided. Amisulpride has not been shown to be better than other SGAs [14]. More data on cariprazine are needed to establish its comparative efficacy in the treatment of predominant and persistent negative symptoms [39].

#### Treatment-resistance and the role of clozapine in the treatment of negative symptoms

Definitions of treatment resistance have been heterogeneous and have initially focused on positive symptoms [46]. However, treatment resistance can also concern other symptom domains. The Treatment Response and Resistance in Psychosis Working Group (TRRIP) has suggested to define treatment resistance with reference to specific symptom domains including positive, negative, and cognitive symptoms. Since the adequate pharmacological treatment with respect to drug, dose, and duration for negative and cognitive symptoms remains to be better defined, the notion of treatment-resistance is often difficult to apply.

Overall, our working group has identified three prototypical clinical situations related to different forms of treatment resistance that should help to clarify the role of clozapine in the treatment of negative symptoms. First, a patient may show negative symptoms that are considered to be secondary to treatment-resistant positive symptoms. In this case, a trial of clozapine is warranted (see section “*Negative symptoms secondary to positive symptoms*” on secondary negative symptoms). Second, the patient may show a broader range of symptoms including negative symptoms that are resistant to treatment. In this case, negative symptoms cannot necessarily be identified as secondary to positive symptoms. Nevertheless, a trial of clozapine is justified with the aim of improving negative symptoms in the context of a global symptom improvement [31,32]. Third, a patient may show primary and persistent or deficit negative symptoms with only limited positive symptoms. This situation corresponds most closely to the definition by the TRRIP of “treatment-resistant schizophrenia-negative symptom domain” [46]. For this type of patient, there is at present no evidence that clozapine is superior to other antipsychotic drugs [39,47].

### Add-on treatment

#### Antidepressants

Add-on of an antidepressant to an antipsychotic has long been considered of potential interest in the treatment of negative symptoms even in the absence of comorbid depression. Two recent meta-analyses of very high quality have summarized the available evidence [25,27] and provide thus the foundation of the present recommendations (see eTable 2). Since these two meta-analyses synthesize all the available evidence, we did not include earlier meta-analyses on the topic.

Helfer et al. included all trials investigating an add-on of an antidepressant to an antipsychotic medication [25]. They found a small beneficial effect for antidepressant add-on on negative symptoms (SMD −0.30, CI −0.44 to −0.16) that was more prominent in a subgroup of studies requiring at least a minimum threshold of negative symptoms (SMD −0.58, CI −0.94 to −0.21).

Galling and colleagues restricted the included studies to those that added the antidepressant to an ongoing antipsychotic therapy and excluded studies that co-initiated both drugs [27]. Their criteria correspond thus to the relevant clinical situation, in which antipsychotic response for negative symptoms is not sufficient and other treatment alternatives are considered. They also found a small beneficial effect of antidepressant add-on on negative symptoms (SMD −0.25, CI −0.44 to −0.06) that was more prominent in a subgroup focusing on negative symptoms as primary outcome (SMD −0.34, CI −0.63 to −0.04). Furthermore, their results did not suggest that the improvement of negative symptoms was secondary to improvement of depressive symptoms. In contrast to improvement of negative symptoms, the authors found no significant improvement of depressive symptoms. Importantly, the original studies were not restricted to patients with primary negative symptoms.

One critical point is their observation that the beneficial effect on negative symptoms was found in studies including patients treated with first-generation antipsychotics, but not in those treated with second-generation antipsychotics. This observation has to be interpreted with caution as only a limited amount of studies was available, in particular concerning studies focusing on negative symptoms, a considerable number of subgroup analyses was conducted, and the increasing placebo response rate might have specifically affected the more recent studies using second-generation antipsychotics.

Finally, the two meta-analyses arrive at somewhat different conclusions concerning the efficacy of particular drugs classes or individual drugs. Galling et al. found Selective Serotonin Reuptake Inhibitors (SSRIs) and Serotonin and Noradrenaline Reuptake Inhibitors (SNRIs) to be superior to placebo for the improvement of negative symptoms, while other antidepressant classes were not. Helfer et al. found negative symptoms improvement for SSRIs, SNRIs, tetracyclic antidepressants (mirtazapine and mianserin), and Monoamine Oxidase Inhibitors (MAOIs). Thus, both meta-analyses conclude on an effect of SSRIs and SNRIs on negative symptoms, although they consider their results exploratory because of the limited number of available studies in particular for SNRIs.

In addition, we searched for randomized controlled trials published after the end date of the search by Galling et al. on October 10, 2017, but we did not find any recent RCT that would change the conclusions drawn from the two discussed meta-analyses.

Overall, with these two meta-analyses, there is now evidence for recommending an antidepressant add-on to antipsychotic medication. However, the Galling et al. meta-analysis corresponds most to situations in which an antidepressant is added to ongoing antipsychotic medication and reports an effect for antidepressant add-on to first-generation but not second-generation antipsychotics with the limitations discussed above. This renders a recommendation for antidepressant add-on complicated, because the working group considered that a patient treated with a first-generation antipsychotic should be switched to second-generation antipsychotic before adding an antidepressant. Nevertheless, due to the limitations regarding the first-generation vs second-generation analysis discussed above, the working group considers that a trial of an antidepressant add-on can be justified independently of the specific ongoing antipsychotic treatment.

Thus, there are many limitations to the generalizability of the results. Importantly, most of the trials included in the meta-analyses did not primarily target negative symptoms and there are questions on which antidepressant to combine with which antipsychotic. Therefore, a grade B recommendation to consider treatment with an antidepressant as an option is provided, although two high-quality meta-analyses are available. Since antidepressant add-on bears the risk of polypharmacy, the decision to add an antidepressant has to be made on an individual basis after careful evaluation of risks and benefits and the antidepressant should be stopped if no improvement is observed. There is still need for at least one large high-quality trial specifically targeting negative symptoms.

***Recommendation 6*** [25–27].

**Table:** 

Grade	Recommendation
B	If negative symptoms do not improve after optimization of antipsychotic treatment, a trial with an add-on antidepressant should be considered for patients with negative symptoms after careful evaluation of risks and benefits. This recommendation concerns also patients who do not show depressive symptoms. However, no specific recommendations for patients with primary negative symptoms can be given. SSRIs are the most studied agents, but the currently available evidence does not allow a recommendation for a particular drug class or individual drug.

***Recommendation 7*** [25–27].

**Table:** 

Grade	Recommendation
B	If the trial with an add-on antidepressant is not associated with an improvement of negative symptoms and/or depression, the antidepressant should be discontinued to avoid polypharmacy.

#### Research on repurposing agents as add-on to antipsychotic therapy

Pro-dopaminergic agents have been studied as potential add-on treatment for negative symptoms. Most randomized controlled trials have been conducted with modafinil and armodafinil [48]. In a recent meta-analysis, Sabé and colleagues did not find a consistent beneficial effect on negative symptoms, and thus no recommendations can be formulated at this point [49].

The N-methyl-D-aspartate (NMDA) receptor antagonist memantine is used off-label in some countries for the treatment of negative symptoms in schizophrenia. Two recent meta-analyses have suggested a beneficial effect [50,51]. However, heterogeneity was very high in both meta-analyses and beneficial effects were mainly driven by few studies conducted outside of Europe with an extremely high effect size [52]. Therefore, no recommendation can be given at this point.

Anti-inflammatory or potential neuroprotective drugs have also been tested in schizophrenia, however, there is no evidence of their efficacy including celecoxib, davunetide, and fatty acids. For some promising “broadly active substances” (e.g., aspirin), we recommend investigations aimed to verify whether their potential beneficial effects in schizophrenia are mediated by their anti-inflammatory properties [53].

As to antibiotics, a recent well-conducted RCT showed no advantage of minocycline over placebo on negative symptoms [54], while a previous meta-analysis of six RCTs [55] found superiority versus placebo for both cognitive deficits and negative symptoms and no advantage on positive symptoms. These findings are preliminary and await further replication.

The prosocial effects of the neuropeptide oxytocin have led to its consideration for the treatment of negative symptoms, but the available evidence is limited and does not support its use for this indication so far [56]. Nutritional supplements and various diets have been intensively tested for psychiatric disorders, syndromes, and symptoms, however no conclusion can be drawn yet about their efficacy [57].

### Brain stimulation

#### Transcranial magnetic stimulation

Noninvasive brain stimulation added to ongoing antipsychotic therapy has received considerable attention as a potential alternative for the treatment of negative symptoms. Several recent meta-analyses of high quality have quantitatively summarized the available literature (see eTables 3 and 4) [58–60].

Only the meta-analysis by Aleman and colleagues focuses on studies specifically targeting negative symptoms that have employed repetitive transcranial magnetic stimulation of the dorsolateral prefrontal cortex. Even after exclusion of two outliers, they reported a beneficial effect of active prefrontal repetitive transcranial magnetic stimulation (rTMS) over sham rTMS (SMD 0.31, CI 0.12–0.50). One critical issue for clinical application of rTMS concerns the stimulation parameters. Although the results have to be considered with caution, the meta-analysis suggests effectiveness in particular for left prefrontal rTMS with a frequency of 10 MHz, intensity above motor threshold, and more than 7,500 stimulations per week. Two other recent meta-analyses show results that are mostly consistent with those obtained by Aleman et al. [59,60].

Across all meta-analyses, inclusion criteria of the original studies were heterogeneous and no conclusions on effects specific for primary or predominant negative symptoms can be drawn. Another concern is the paucity of follow-up data after the end of the intervention, which precludes any conclusions on the persistence of the effect. In addition, the largest high-quality RCT, included in the meta-analysis, did not find a beneficial effect of left prefrontal rTMS on negative symptoms [61].

Side effects of rTMS were not addressed in the meta-analyses. rTMS is generally considered to have a benign side-effect profile [62] and the largest multicenter trial did not report a higher rate of side effects in the active TMS group [61]. However, Kennedy et al. reported a nonsignificant worsening of positive symptoms that became significant in subgroup analyses with stimulation frequency >20 Hz, stimulation intensity >110%, trials lasting over 3 weeks, and treatment site over the left prefrontal cortex [59]. Although these results do not allow to conclude on a safety issue, more information on potential side effects is needed in relation to the relatively high stimulation intensity and frequency suggested to be effective against negative symptoms. Another important point concerns the fact that application of rTMS requires considerable expertise and most considered studies have been conducted in expert centers. The real-world effectiveness of rTMS for negative symptoms thus remains an open issue.

Overall, the working group considered rTMS as a very promising approach to the treatment of negative symptoms, but did not consider the available evidence sufficient for a general recommendation. However, in expert centers, treatment with left prefrontal rTMS can be considered for patients with negative symptoms that do not improve with other interventions.

#### Transcranial direct current stimulation

The meta-analyses discussed in the previous section have also addressed transcranial direct current stimulation (tDCS), which is another method for noninvasive brain stimulation. Overall, these meta-analyses suggest that a moderate effect on negative symptoms in comparison with sham tDCS [59,60], but the effect is not always significant [58]. The number of available studies and included patients is small and studies have rarely targeted negative symptoms as a primary outcome. Therefore, the available meta-analyses do not allow formulating any recommendations at this point. In addition, we searched for randomized controlled trials published after the end date of the search by Aleman et al. on December 31, 2017, but we did not find any recent RCT that would change the decision not to formulate a recommendation.

#### Other brain stimulation techniques

The evidence for other brain stimulation approaches for the treatment of negative symptoms remains very limited. While electroconvulsive therapy has shown promising effects on overall and positive symptoms in treatment-resistant schizophrenia, the evidence for a reduction of negative symptoms [63–65] is very limited and no recommendation can be given at this point.

Invasive deep-brain stimulation has so far received little attention for the treatment of schizophrenia. With respect to negative symptoms, a stimulation of potentially hypoactive regions including the ventral tegmental area and the nucleus accumbens is of potential interest, but a clinical trial targeting these regions was not able to include any patient (https://clinicaltrials.gov/ct2/show/NCT01725334). No recommendation can be given at this point.

## Psychosocial Treatments

### Psychotherapy

#### Cognitive behavior therapy

The anticipation of negative symptoms to some degree being a result of the individual lacking the ability and power to tackle different life situations forms the foundation for examining whether cognitive behavioral therapy could ameliorate negative symptoms. A better understanding of the nature of negative symptoms could be helpful in shaping future treatment.

There is some evidence that CBT is an effective treatment for psychotic symptoms in schizophrenia, and most studies of CBT in schizophrenia have focused on the treatment of psychotic symptoms. This has increased the hopes that CBT also could prove to be an effective treatment of negative symptoms.

Based on randomized clinical trials, several meta-analyses have examined the effect of CBT on negative symptoms (eTable 5). Most of them conclude that there is only modest or no effect on negative symptoms, but many of the studies included in the meta-analyses did not include patients with severe negative symptoms, nor did they primarily aim to reduce negative symptoms. Different techniques are needed to treat negative or psychotic symptoms.

Jauhar et al. conducted a meta-analysis based on 34 randomized clinical trials and examined the effect of CBT on negative symptoms among other outcomes. The authors identified a small, but significant effect (g −0.13, CI −0.25 to −0.01), which they considered to be of questionable clinical significance [34].

Lutgens and colleagues also included CBT in a meta-analysis addressing a wider range of interventions and found a small but significant effect (SMD −0.34, CI −0.55 to −0.12) [66]. However, they did not identify all available trials and the inclusion of “negative symptoms” in the search term might bias toward the identification of studies with stronger effects.

The most comprehensive meta-analysis was conducted by Velthorst et al. [67]. In that paper, the studies were divided into studies with negative symptoms as a secondary or primary outcome, respectively. They identified 30 randomized clinical trials. There were no significant effects on negative symptoms, neither in studies with negative symptoms as secondary nor as primary outcome (g 0.093, CI −0.028 to 0.214 and g 0.157, CI −0.10 to 0.409). However, it is important to note that only two studies specifically targeting negative symptoms were available. Meta-regression showed that positive effects were associated with earlier year of publication.

One RCT, not included in the abovementioned meta-analyses, targeted functional outcome as assessed with the Global Assessment Scale [68]. In addition to improvement on the Global Assessment Scale, the authors found a significant effect on avolition/apathy, but not on anhedonia/asociality, alogia, and affective flattening. A recent RCT investigated a group program specifically targeting apathy and anhedonia and found an improvement at the end of the intervention at follow-up [69]. Thus, these CBT interventions specifically targeting negative symptoms have shown promise, but no additional recent trials were identified that would change the conclusions from the Velthorst et al. meta-analysis at this point.

Based on the existing literature, the evidence base for recommending CBT for the treatment of negative symptoms is not sufficient to formulate a recommendation. Most meta-analyses have found small or insignificant effects of CBT on negative symptoms. This can partly be explained by only few studies having high level of negative symptoms as an inclusion criterion, and negative symptoms as the main focus of treatment. While there are promising results from two recent trials targeting negative symptoms, these trials do not yet allow changing the conclusions from the meta-analyses discussed and providing a recommendation. It is suggested to carry out at least one large high-quality trial with inclusion and outcome criteria focusing primarily on negative symptoms.

#### Body-oriented and mind–body psychotherapies

Another group of therapies has employed body-oriented or mind–body approaches. While initially there were encouraging results for body-oriented psychotherapies, a recent multicenter trial did not find a beneficial effect on negative symptoms [70]. There is no clear definition for mind–body therapies. In a recent meta-analysis, Sabé and colleagues found a beneficial effect of mindfulness-based therapies on negative symptoms [71], but data were available for only three studies not specifically targeting negative symptoms. Therefore, the available evidence does not allow any recommendation, but an increasing number of studies is being conducted on these approaches.

#### Art therapy and music therapy

The National Institute for Health and Care Excellence (NICE) guidance recommends consideration of art therapies for all people with psychosis or schizophrenia, in particular for improving negative symptoms [72]. This recommendation has been criticized as having an insufficient evidence base [73]. In addition, NICE employs a broad definition of art therapies that includes body-oriented psychotherapies discussed above as well as art therapy in the strict sense and music therapy. The evidence for art therapy has recently been reviewed by Attard who concluded that the evidence is currently inconclusive [74]. We found no more recent studies that would change this conclusion.

Music therapy has been more intensively studied than fine art therapy. A recent Cochrane review has concluded that there is evidence for a beneficial effect of music therapy on mental state, including negative symptoms [75]. However, it is a matter of debate whether the included studies provide sufficient evidence for a European recommendation. For example, for short-term results on negative symptoms, data from five studies were available. The three studies that drove the observed beneficial effects were conducted with inpatient populations in China and Iran. Therefore, music therapy seems to have a potential for improving negative symptoms, but a larger trial including outpatients would be needed before a recommendation can be given.

### Training interventions

#### Social skills training

Social skills training is a psychosocial intervention focusing on improvement of social interaction and interpersonal skills. Social skills training involves role plays, coaching, and feedback. Social skills training was originally developed for chronic patients in the era of deinstitutionalization, with a strong focus on very basic skills. More recently, Early Intervention Services have modified it, and the treatment has also been shown to be beneficial for patients with less severe conditions. The modern versions of social skills training also include approaches focused primarily on social cognition, and there is also resemblance to cognitive-behavioral techniques. Moreover, psycho-education, life management skills, and relapse prevention strategies have been embraced by the term social skills training.

Several meta-analyses have addressed the effectiveness of social skills training on negative symptoms (eTable 6). In a meta-analysis conducted by Lutgens et al. [66], the authors found a moderate effect on negative symptoms (SMD 0.44, CI 0.10–0.77). The meta-analysis included a total of 17 RCTs using skills training (*n* = 11), occupational therapy (*n* = 3), cognitive adaptation training (*n* = 2), or vocational training (*n* = 1). Thus, a broader spectrum of studies was included and not all studies in the skills training subgroup specifically targeted social skills. Nevertheless, a subgroup analysis for the skills training studies showed a beneficial effect on negative symptoms. Overall, the effects were stronger when compared to treatment as usual (TAU) than when compared to active controls.

The most recent meta-analysis by Turner et al. [76] provided a systematic and very comprehensive overview of the efficacy of social skills training for psychosis. The authors found that social skills training demonstrated superiority for negative symptoms against all comparators pooled (g 0.191, CI 0.043–0.33), against treatment as usual (g 0.311, CI 0.078–0.544), and against active controls (for high-quality studies only g 0.196, CI 0.010–0.383), with small but reliable differences. A subgroup analysis addressing different subtypes of social skills training was limited by the small number of studies available for each intervention. It is important to note that no differentiation between primary and secondary negative symptoms was conducted.

Even though some studies had a very low power, and in some negative symptoms were not the primary outcome, it seems convincing that social skills training is superior to both treatments as usual and to active comparators. Therefore, social skills training should be recommended for treatment of patients with psychosis and negative symptoms. The available evidence does not yet allow to recommend a specific type of social skills training.

***Recommendation 8*** [66,76]

**Table:** 

Grade	Recommendation
B	Social skills training should be offered to patients with negative symptoms, but no specific recommendation for patients with primary negative symptoms can be given. Furthermore, the available evidence does not allow recommending one specific program for social skills training.

#### Cognitive remediation

Cognitive remediation was developed to target cognitive impairments, which, like negative symptoms, are a major predictor of functional outcome. Cognitive remediation employs and combines different approaches such as repeated task performance, feedback, and development of compensatory strategies. Assessment and treatment of cognitive impairment are the topic of another EPA guidance developed in parallel to the present recommendations.

Earlier meta-analyses did not report the effect of cognitive remediation on negative symptoms [77]. However, a recent high-quality meta-analysis found a beneficial effect of cognitive remediation on negative symptoms post-treatment (g 0.30, CI 0.22–0.36) and after a variable follow-up (g 0.36, CI 0.21–0.51) [78]. Cognitive remediation was superior to TAU and to active control conditions. However, it has to be kept in mind that studies on cognitive remediation generally do not target negative symptoms as the primary outcome. In addition, the interventions subsumed under the term cognitive remediation are very heterogeneous in terms of trained cognitive functions, the used tools, and additional components added to cognitive training.

We applied the search criteria employed by Cella and colleagues (eTable 7) to search for additional RCTs that have appeared after the inclusion period of their meta-analysis. No RCT likely to change the main conclusions from their meta-analysis was identified. Cella and colleagues note that the included high-quality studies with the strongest effect size all have additional components such as supported employment or practicing trained skills in everyday life [78]. It is therefore very difficult to give a precise recommendation on the use of cognitive remediation specifically for the treatment of negative symptoms. At least one high-quality RCT specifically targeting negative symptoms with cognitive remediation should be conducted, ideally allowing to identify the relevant components of the intervention. However, it is worth considering cognitive remediation for patients with negative symptoms, in particular for those who also show cognitive impairment.

***Recommendation 9*** [78]

**Table:** 

Grade	Recommendation
C	Cognitive remediation can be considered for patients with negative symptoms, in particular for those who also show cognitive impairment. No specific recommendation for cognitive remediation in the treatment of primary or predominant negative symptoms can be given.

### Exercise

Exercise, and in particular aerobic exercise, has been suggested to improve negative symptoms (eTable 8). In a meta-analysis, Dauwan and colleagues showed broad beneficial effects of a heterogeneous set of exercise interventions on negative symptoms, but also on other symptom dimensions, quality of life, and global functioning [79]. A recent meta-analysis by Vogel and colleagues focused on negative symptoms and found a beneficial effect across a broad range of interventions including mind–body and aerobic exercise [80]. Sabé and colleagues found a beneficial effect specifically for aerobic exercise [81].

It has to be acknowledged that few of the original studies focused on negative symptoms as the primary outcome and no conclusions about the effects on primary or predominant negative symptoms can be drawn. In addition, the risk of bias was high in most studies. Therefore, the evidence for a specific recommendation of exercise for the treatment of negative symptoms remains limited and is indirect.

However, the present recommendation has to be evaluated in the context of evidence for the improvement of physical health with exercise and existing recommendations for physical activity [82]. Therefore, we cannot specifically recommend exercise for the treatment of negative symptoms, but recommend considering exercise as part of a treatment package also aiming at improving the physical health for persons with negative symptoms. Although aerobic exercise might be particularly beneficial, the available overall evidence does not allow recommending one form of exercise over another.

***Recommendation 10*** [79–81]

**Table:** 

Grade	Recommendation
C	Exercise can be considered for persons suffering from negative symptoms as part of an integrated treatment plan also aiming at improving physical health.

### Access to treatment and psychosocial rehabilitation

Patients with negative symptoms often show reduced motivation in particular in the social domain. This can also limit their access to treatment, if they are required to actively come to an outpatient clinic.

The existing data on community treatments do not provide evidence for specific interventions for patients with negative symptoms [83]. However, the guidance group considers low-threshold—including assertive community—interventions to be useful in improving the access of patients with negative symptoms to psychiatric care. Although the lack of specific evidence for people with negative symptoms would be compatible with a grade D recommendation, all members of the EPA guidance group on negative symptoms agreed to upgrade the recommendation to grade B based on their knowledge and experience.

***Recommendation 11*** [83]

**Table:** 

Grade	Recommendation
B	The access to care for patients with negative symptoms should have a low-threshold and should be facilitated by assertive community interventions.

For patients with negative symptoms, both motivational and expressive deficits can limit their access to work and leisure activities as well as adequate living conditions. Trials of rehabilitation interventions have not specifically targeted negative symptoms [84,85]. Nevertheless, the guidance group considers that an effort is necessary to ensure the access of patients with negative symptoms to rehabilitation interventions including supported employment and supported housing. Although the lack of specific evidence for people with negative symptoms would be compatible with a grade D recommendation, all members of the EPA guidance group on negative symptoms agreed to upgrade the recommendation to grade B based on their knowledge and experience.

***Recommendation 12*** [84,85]

**Table:** 

Grade	Recommendation
B	Patients with negative symptoms should have access to rehabilitation interventions such as supported employment and supported housing.

### General comment on psychosocial interventions

Across the different psychosocial interventions, most of the studies included in the different meta-analyses did not specifically target negative symptoms and few studies defined a minimum threshold for negative symptoms at inclusion. Future studies in this field should have negative symptoms as the primary outcome, and the content of the treatment should be specifically focused on how to tackle negative symptoms and their effect on daily life.

Several sources of heterogeneity other than the primary outcome need to be considered. Studies in the meta-analyses were very different with regard to duration of treatment, which makes comparisons difficult. Most of the studies in the meta-analyses compared the respective psychosocial intervention to treatment as usual. This approach can be justified, if the purpose of the study is to prove whether the intervention is better than usual practice. However, if the research question is whether the intervention is better than other psychosocial treatments, the study should include an active comparator. Furthermore, several studies combined more than one psychosocial intervention [86], which makes it difficult to disentangle the specific effects. Finally, patient populations were heterogeneous, but many of the studies in the meta-analyses were conducted in patients who have been ill for many years and may have difficulties in changing behavior. It is important, and possibly more promising, to carry out trials in patient populations in early phases of their illness.

Many of the studies included in the meta-analyses had a small number of participants. Although meta-analyses can counteract this limitation to some degree, studies should preferably be sufficiently powered to identify significant effects on negative symptoms. Some authors found earlier studies to have better outcome than later studies [67]. This is most likely due to better quality of recent studies with more strict demands for blinding of assessments, concealed treatment allocation sequence, well-described randomization procedure, and preplanned outcome as recommended in the extended Consolidated Standards of Reporting Trials (CONSORT) criteria [87].

Despite these difficulties, recent research on the effects of psychosocial interventions is promising, although the currently available evidence only allows limited recommendations. Regarding the problems discussed above, even the limited recommendations for social skills training, cognitive remediation, and exercise can be criticized. However, we would like to note that, as psychosocial interventions have a favorable benefit/risk ratio, we applied somewhat less strict efficacy criteria than for biological interventions.

## Specific Treatments Depending on Illness-Stage

### At-risk mental-state

Negative symptoms are frequently present in persons with an at-risk mental-state. They have an important impact on functioning and predict conversion to psychosis. Therefore, effective treatment of negative symptoms in this population is of high interest. Devoe and colleagues have recently published a comprehensive meta-analysis including a wide range of psychosocial and pharmacological intervention studies [88]. They did not find evidence of effects on negative symptoms for any of the included treatment approaches and note that none of the included studies primarily targeted negative symptoms.

### First-episode psychosis

For several decades, Early Intervention Services have been a central pillar of treatment of all first-episode psychosis. Specialized assertive early intervention includes intensive and assertive case management with frequent contact with a staff member in a multidisciplinary team, family involvement, and recovery-oriented group programs. The concept was first implemented in Australia, United Kingdom, and Canada and later evaluated in randomized clinical trials, first in the United Kingdom [89], soon after in Denmark [90], and later in several other countries [91]. The approach involves cognitive therapy-based case-management which focuses on strategies to overcome daily hassles and to identify islands of engagement and volition. Many patients will not have enough power to force through the barriers for getting treatment and social support.

The meta-analysis by Correll et al. [91] demonstrated that across the trials there was a modest but consistent effect on negative symptoms (SMD 0.28, CI 0.14–0.42). However, an amelioration with comparable effect size was observed for positive, depressive, and general psychopathology. A more robust effect on negative symptoms was observed for two large trials: OPUS [90] and Recovery after an initial episode of schizophrenia - early treatment program (RAISE-ETP) [92], in which the effect was stronger for negative than for positive symptoms.

Future studies or meta-analyses should attempt to focus on primary and persistent negative symptoms.

***Recommendation 13*** [91]

**Table:** 

Grade	Recommendation
B	Early intervention services should be provided for patients with a first episode of psychosis. There is evidence that the use of these services can improve negative symptoms. However, no specific recommendation can be formulated with regard to the type of intervention program and the target population with primary and persistent negative symptoms.

Please note that the working group members consider that the recommendation to treat patients with first-episode psychosis with second-generation rather than first-generation antipsychotics [93] is also relevant for avoiding secondary negative symptoms due to medication side effects.

## Discussion

Based on the evaluation of the data available on the treatment of negative symptoms in schizophrenia, the authors could identify both definitive progress and need for further research in this field.

There is clearly a lack of high level or even sufficient evidence about the efficacy and comparative effectiveness of various interventions. This has been an important limitation in the development of this guidance as most original studies did not even specify negative symptoms as their primary outcome. Even where high-level evidence was available, extrapolation was needed to some degree, which results in the absence of any grade A recommendation.

Importantly, we were unable to provide any specific recommendations for the treatment of primary or predominant negative symptoms. Many well-designed original studies and meta-analyses did neither differentiate between primary and secondary negative symptoms nor did they analyze persistent and/or predominant negative symptoms. Current research has been focusing on the persistence of negative symptoms rather than on their predominance, since predominance of positive or negative symptoms may vary across time, and therefore represent a temporary characteristic of the disorder. This is not reflected by the European regulatory requirements [37]. The current definitions of negative symptoms have evolved and are different from the ones used in earlier treatment studies [9].

All of these issues contributed to the decision not to provide specific recommendations for primary, predominant, or persistent negative symptoms. However, some progress can be highlighted.

Regarding antipsychotics, the results for amisulpride and cariprazine are promising, although we considered further research to be necessary before giving a recommendation. As to antidepressants, we now have support for using them in the treatment of negative symptoms in clinical practice, but no specific recommendation for primary negative symptoms can be given.

As described above, a number of specific psychosocial interventions have shown promise. Social skills training has been found to be better than TAU in the treatment of general negative symptoms. Exercise-based interventions and cognitive remediation can be considered in the context of broader treatment targets. Other interventions such as CBT, mindfulness-based therapy, and art therapies are promising but trials specifically targeting negative symptoms are needed. Importantly, the working group considers it important to ensure the access of patients with negative symptoms to treatment and to psychosocial rehabilitation.

Last but not least, we would like to encourage active treatment of the early phase of schizophrenia: active treatment in the early phase is associated with better course and outcomes of schizophrenia, and there is evidence that early intervention services can improve negative symptoms.

Overall, due to the very small number of studies specifically targeting negative symptoms as a primary outcome, the evidence base can evolve rapidly with the publication of a large well-designed RCT targeting negative symptoms. We therefore encourage the readers to follow the evolution of the literature when using the present recommendations.

## Conclusions

Treatment of negative symptoms in schizophrenia remains a major unmet need.

There is a lack of high-level evidence supporting specific interventions. However, some pharmacological and non-pharmacological treatment methods have been found to be effective in the treatment of patients with undifferentiated negative symptoms of schizophrenia.

Every effort should be made to use the available interventions for their treatment, since even minor improvement in negative symptoms may be associated with better functioning and better quality of life. Therefore, an effort should be made to ensure access of patients not only to the recommended interventions, but also to treatment and to psychosocial rehabilitation in a broad sense.

In the light of the different biological and psychosocial treatment options, treatment should be personalized and guided by the individual patient’s preferences.

Negative symptoms of schizophrenia need significantly more research, in particular on the treatment of persistent negative symptoms. We hope that this research will require an update of these recommendations soon.

## Supplementary material

For supplementary material accompanying this paper visit http://dx.doi.org/10.1192/j.eurpsy.2021.13. click here to view supplementary material
