# Supplementary material for: EPA guidance on treatment of negative symptoms in schizophrenia
Source: Eur Psychiatry. 2021 Mar 17;64(1):e21. doi: 10.1192/j.eurpsy.2021.13 (PMC8057437; doi:10.1192/j.eurpsy.2021.13)
Supplement: Supplementary file 1 [file S0924933821000134sup001.docx]

**Index**

- **eTable 1. Evidence for treatment with antipsychotic medication**
- **eTable 2. Evidence for treatment with add-on antidepressant**
- **eTable 3. Evidence for treatment with repetitive transcranial magnetic stimulation**
- **eTable 4. Evidence for treatment with transcranial direct current stimulation**
- **eTable 5. Evidence for cognitive behavior therapy**
- **eTable 6. Evidence for social skills training**
- **eTable 7. Evidence for cognitive remediation**
- **eTable 8. Evidence for physical exercise**
- **References**

**eTable 1. Evidence for treatment with antipsychotic medication.**

| **Study** | **Type of Study** | **Methods^1^** | **Results^2^** | **Limitations^3^** | **Evidence Level** |  |
| --- | --- | --- | --- | --- | --- | --- |
| Leucht, et al., 2017 [1] | Meta-analysis of placebo controlled RCTs | P: Patients with acute exacerbations of schizophrenia or related disorders  I: All antipsychotics licensed in at least one country, except clozapine. Duration of at least 3 weeks.  C: Placebo  O: Change in overall symptoms. Change in negative symptoms measured with PANSS or BPRS was a secondary outcome. | Primary outcome: The mean effect size for overall efficacy of all studies combined was 0.47 (CI 0.42 to 0.51; 105 studies, N=22’741 participants).  Secondary outcome: Effects on negative symptoms were small but significant (SMD 0.35, CI 0.31 to 0.40; 69 studies, N=18’632). | Studies in patients with predominant negative symptoms were excluded.  Limitation to studies of acute exacerbations limits relevance for negative symptoms.  All antipsychotics were analyzed as a class. | I | |
| Galling, et al., 2017 [2] | Meta-analysis of randomized trials | P: Patients with schizophrenia or schizoaffective disorder  I: Augmentation with a second antipsychotic  C: Placebo + continued antipsychotic monotherapy  O: Total symptom reduction and study-defined response. Negative symptom changes measured with SANS, PANSS or BPRS were considered as secondary outcome. | Primary outcome: Antipsychotic augmentation was superior to monotherapy regarding total symptom reduction (SMD -0.53, CI -0.87 to -0.19; 16 studies, N=694). However, superiority was not apparent in double-blind and high-quality studies. Study-defined response was not significantly different between antipsychotic augmentation and monotherapy (RR 1.19, CI 0.99 to 1.42; 14 studies, N=938), being clearly non-significant in double-blind and high-quality studies.  Secondary outcome: Negative symptoms improved more with augmentation treatment (SMD -0.38, CI -0.63 to -0.13; 18 studies, N=931), but only in studies augmenting D2 antagonist with a partial agonist (SMD -0.41, CI -0.79 to -0.03; 8 studies, N=532). | Limited number of studies, in particular those of high quality and those reporting negative symptoms.  Heterogeneity of study designs. For example, heterogeneous outcomes between efficacy and adverse effect targets.  No information on patients with primary, predominant or prominent negative symptoms.  Authors report 11 studies on partial agonist analysis, but do not specify which were the 8 studies included in negative symptom analysis. | I | |
| Krause, et al., 2018 [3] | Meta-analysis of randomized trials (separate pairwise meta-analysis) | P: Patients with schizophrenia, schizophreniform disorder, or schizoaffective disorder with predominant or prominent negative symptoms  I: All second-generation antipsychotics available in the US and/or Europe, and a selection of first-generation antipsychotics  C: Placebo or another antipsychotic  O: Mean change of negative symptoms from baseline to endpoint measured with any validated scale. | Primary outcome: In patients with predominant negative symptoms amisulpride was superior to placebo (SMD 0.47, CI 0.23 to 0.71; 4 studies, N=590)  Cariprazine was superior to risperidone (SMD −0.29, CI −0.48 to −0.11; 1 study, N=456). Olanzapine was superior to haloperidol in a small trial (SMD 0.75, CI 0.06 to 1.44; 1 study, N=35).  In patients with prominent negative symptoms, olanzapine and quetiapine were superior to risperidone in single trials. Overall, studies in prominent negative symptoms were potentially more confounded by improvements of secondary negative symptoms. | Overall, very few sufficiently powered studies available.  In several studies of patients with prominent negative symptoms, the effect sizes for positive symptoms were either identical or higher than those for negative symptoms. This renders the possibility of pseudospecificity more likely than for the studies on predominant negative symptoms.  The only cariprazine study was sponsored by the manufacturer and placebo-controlled evidence is lacking. | I | |
| Huhn, et al., 2019 [4] | Network meta-analysis of placebo-controlled and head-to-headRCTs | P: Patients with acute exacerbations of schizophrenia or related disorders  I: All second-generation (atypical) antipsychotics available in Europe or the USA and a selection of first-generation antipsychotics  C: Placebo or other antipsychotics  O: Change in overall symptoms. Change in negative symptoms measured with any published scale was a secondary outcome. | Primary outcome: 26 of 32 antipsychotics significantly improved overall symptoms compared to placebo (218 studies, N=40’815). Clozapine, amisulpride, zotepine, olanzapine and risperidone were associated with stronger improvement than many other drugs.  Secondary outcome: 18 of 21 antipsychotics significantly improved negative symptoms compared to placebo (132 studies, N=32’015). SMDs for antipsychotics that significantly reduced negative symptoms compared with placebo ranged between -0.62 (CI-0.84 to -0.39) for clozapine to -0.22 (CI -0.33 to -0.11) for iloperidone. Clozapine, amisulpride, olanzapine, and, to a lesser extent, zotepine and risperidone were associated with a stronger improvement of negative symptoms significantly than many other drugs. | Studies in patients with predominant negative symptoms were excluded.  Limitation to studies of acute exacerbations limits relevance for negative symptoms.  The included studies were conducted over a 60-year period, during which study characteristics changed.  Threat to the transitivity assumption of the conducted network meta-analysis as the placebo response has increased over the years. | I | |

^1^Methods are described following the PICO schema - (P)opulation, (I)ntervention, (C)omparator, (O)utcomes.

^2^For the results only the primary outcome and all secondary outcomes relating to negative symptoms are reported.

^3^Only limitations potentially concerning the interpretation of the results for negative symptoms are reported.

**eTable 2. Evidence for treatment with add-on antidepressant.**

| **Study** | **Type of Study** | **Methods** | **Results** | **Limitations** | **Evidence Level** |
| --- | --- | --- | --- | --- | --- |
| Helfer, et al., 2016 [5] | Meta-analysis of randomized controlled trials | P: Patient with schizophrenia or schizophrenia (-like) psychosis  I: Add-on of antidepressant to antipsychotic treatment for at least one week of duration  C: Placebo or no intervention added to antipsychotic  O: Depressive symptoms and negative symptoms measured with SANS, PANSS or BPRS were co-primary outcomes. | Primary outcomes: Add-on antidepressants appeared more efficacious than controls for depressive symptoms (SMD -0.25, CI -0.38 to -0.12; 42 studies, N=1849), and for negative symptoms (SMD -0.30, CI -0.44 to -0.16; 48 studies, N=1905).  Relevant additional analyses:  The effects on depressive and negative symptoms appeared more pronounced in studies requiring severity above a threshold (SMD -0.34, CI -0.58 to -0.09; 5 studies, N=330, and SMD -0.58, CI-0.94 to -0.21; 10 studies, N=406 respectively).  Negative symptom improvement observed for SSRIs, SNRIs, tetracyclic antidepressants (mirtazapine and mianserin) and MAO-inhibitors | Many small studies were included, exposing to an inflated effect sizes  Original studies did not target primary or predominant negative symptoms  Very limited number of studies for each AP / antidepressant combination | I |
| Gregory, et al., 2017 [6] | Meta-analysis of randomized controlled trials | P: Schizophrenia or related psychosis  I: Antidepressant (in all studies as add-on to antipsychotic therapy)  C: Usual care or placebo  O: Response as dichotomous outcome, change on standardized depression rating scale as continuous outcome | Primary outcomes: Antidepressant favored over control for response (Risk difference -0.19, CI -0.27 to -0.11; 8 studies, N=515).  Secondary outcomes: Main analysis antidepressant favored over control for depression scores (SMD -0.24, CI -0.48 to -0.01; 17 studies, N=879), but results become non-significant after exclusion of one outlier study with very large effect size. | Negative symptoms not reported as outcome  Heterogeneity of original studies  Very limited number of studies for each antidepressant | I |
| Galling, et al., 2018 [7] | Meta-analysis of randomized controlled trials | P: Primary diagnoses of schizophrenia (<50% schizoaffective, schizophreniform or delusional disorder)  I: Add-on of antidepressant to ongoing antipsychotic treatment of at least two weeks duration before inclusion.  C: Add-on of placebo to ongoing antipsychotic  O: Primary outcome total psychopathology. Secondary outcomes positive, negative (PANSS, BPRS, SANS) and depressive symptoms. | Primary outcome: Antidepressant favored over placebo for total symptoms (SMD 0.37, CI 0.57 to 0.17; 30 studies, N=1311)  Secondary outcomes: Antidepressant favored over placebo for negative symptoms (SMD 0.25, CI 0.44 to 0.06, p= 0.010, 32 studies, N= 1348). No differences between antidepressants for positive and depressive symptoms  Additional analyses:  Superiority for negative symptoms in studies focusing on total and negative symptoms, but not on depressive symptoms  Superiority for negative symptoms in studies with FGAs but not SGAs  Superiority for SSRIs and SNRIs but not other antidepressant classes | Heterogeneity of original studies  Original studies did not target primary or predominant negative symptoms  Very limited number of studies for each AP / antidepressant combination | I |

**eTable 3. Evidence for treatment with repetitive transcranial magnetic stimulation TMS (rTMS).**

| **Study** | **Type of Study** | **Methods** | **Results** | **Limitations** | **Evidence Level** |
| --- | --- | --- | --- | --- | --- |
| Aleman, et al., 2018 [8] | Meta-analysis of randomized controlled trials | P: Patients with schizophrenia, schizophreniform or schizoaffective disorder  I: rTMS of prefrontal cortex  C: Sham TMS  O: Negative symptoms measured with SANS, PANSS or BPRS | Primary outcome: Favors rTMS over sham (SMD 0.64, CI 0.32 to 0.96; 22 studies, N=825).  After exclusion of two outliers: Favors rTMS over sham (SMD 0.31, CI 0.12 to 0.50; 18 studies, N=721).  Secondary analyses suggest rTMS to be more effective with younger age. The following stimulation parameters are associated with a favorable effect: Left prefrontal rTMS with a frequency of 10 MHz, intensity above motor threshold, more than 7500 stimulations per week | Heterogeneity of the original studies.  No conclusions on effects specific for primary or predominant negative symptoms can be drawn.  The restriction of the search to studies including ‘negative symptoms’ in title, abstract or keywords might introduce a bias for positive results. | I |
| Kennedy, et al., 2018 [9] | Meta-analysis of randomized controlled trials | P: Patients with schizophrenia and related psychoses  I: rTMS  C: Sham stimulation  O: Several outcomes including reduction in auditory hallucinations, positive and negative symptoms (measured with the PANSS) | Compared to sham, rTMS improved hallucinations (Hedge’s g -0.51, CI not reported, p < 0.001; 18 studies N=578) and negative symptoms (g -0.49, CI not reported, p= 0.01; 21 studies, N=869). There was small, non-significant worsening of positive symptoms (g 0.28, CI not reported, p= 0.13; 27 studies, N=999).  Additional outcomes: Higher pulse frequency (>10 Hz), motor threshold intensity of 110%, left prefrontal cortical treatment site and trial duration over 3 weeks were associated with improvement in negative symptoms and worsening in positive symptoms (all p< 0.03). | Heterogeneity of the original studies including stimulation site.  No conclusions on effects specific for primary or predominant negative symptoms can be drawn. | I |
| Osoegawa, et al., 2018 [10] | Meta-analysis of randomized controlled trials | P: Patients with schizophrenia spectrum and other psychotic disorders  I: rTMS (without restriction to a specific stimulation site)  C: Sham TMS  O: Negative symptoms measured with SANS, PANSS or BPRS | Primary outcome: rTMS was superior to sham for the treatment of negative symptoms (g 0.19, CI 0.07 to 0.32; 24 studies, N=1086).  Secondary analyses: Meta-regression showed no significant influence of any variable on the results (number of weeks, of sessions, duration of sessions, frequency and site of stimulation, pulses per session, total number of pulses) | Heterogeneity of the original studies including stimulation site.  No conclusions on effects specific for primary or predominant negative symptoms can be drawn.  Depression scores were not recorded or controlled in the included studies, | I |

**eTable 4. Evidence for treatment with transcranial direct current stimulation (tDCS).**

| **Study** | **Type of Study** | **Methods** | **Results** | **Limitations** | **Evidence Level** |
| --- | --- | --- | --- | --- | --- |
| Aleman, et al., 2018 [8] | Meta-analysis of randomized controlled trials | P: Patients with schizophrenia, schizophreniform or schizoaffective disorder  I: tDCS of the prefrontal cortex  C: Sham stimulation  O: Negative symptoms measured with SANS, PANSS or BPRS | Primary outcome: tDCS no significantly superior to sham stimulation (SMD 0.5, CI -0.07 to 1.07; 5 studies, N= 134)  No secondary analyses possible with tDCS studies due to the small amount of studies. | Small number of tDCS studies  Small size of each primary study.  Heterogeneity of the original studies.  No conclusions on effects specific for primary or predominant negative symptoms can be drawn.  The restriction of the search to studies including ‘negative symptoms’ in title, abstract or keywords might introduce a bias for positive results. | I |
| Kennedy, et al., 2018 [9] | Meta-analysis of randomized controlled trials | P: Patients with patients with schizophrenia and related psychoses  I: tDCS  C: Sham stimulation  O: Auditory hallucinations, positive and negative symptoms measured with the Auditory Hallucinations Rating Scale (AHRS) and/or the PANSS | Primary outcome: Compared to sham, tDCS improved negative symptoms (Hedge’s g -0.63, CI not reported, p= 0.02; 7 studies, N=190), but not auditory hallucinations or general positive symptoms. | Small number of studies.  Small size of each primary study.  Heterogeneity of the original studies.  No conclusions on effects specific for primary or predominant negative symptoms can be drawn.  and the total number of studies, especially for tDCS. | I |
| Osoegawa, et al., 2018 [10] | Meta-analysis of randomized controlled trials | P: Patients with schizophrenia spectrum and other psychotic disorders  I: tDCS (without restriction to a specific stimulation site)  C: Sham stimulation  O: Negative symptoms measured with SANS, PANSS or BPRS | Primary outcome: Active tDCS was superior to sham for the treatment of negative symptoms in schizophrenia (g 0.5, CI 0.02 to 0.97; 7 studies, N=169).  Secondary analyses: Meta-regression showed no influence of any variable on the results (number of weeks, of sessions, duration of sessions, frequency and site of stimulation, pulses per session, total number of pulses) | Small number of tDCS studies.  Small size of each primary study.  Heterogeneity of the original studies including stimulation site.  No conclusions on effects specific for primary or predominant negative symptoms can be drawn.  Depression scores were not recorded or controlled in the included studies. | I |

**eTable 5. Evidence for cognitive behavior therapy (CBT).**

| **Study** | **Type of Study** | **Methods** | **Results** | **Limitations** | **Evidence Level** |
| --- | --- | --- | --- | --- | --- |
| Jauhar, et al., 2014 [11] | Meta-analysis of randomized controlled trials | P: Trials were included if a majority of their patients had a diagnosis of schizophrenia, schizoaffective or non-affective functional psychosis  I: Interventions using individual or group CBT. CBT interventions had to be directed to at least one class of schizophrenia symptoms.  C: Parallel control group of any type (waitlist, TAU or active control) an intervention designed  O: End-of study data on overall, positive and negative symptoms. | Primary outcome: Pooled effect sizes were for overall symptoms (g -0.33, CI -0.47 to -0.19; 34 studies), for positive symptoms (g -0.25, CI -0.37 to -0.13; 33 studies), and in negative symptoms (g -0.13, CI -0.25 to -0.01; 34 studies).  The CBT vs TAU showed beneficial effect on negative symptoms (g -0.17, CI -0.33 to -0.02, 20 studies), whereas, CBT vs control interventions shows no specific effect on negative symptoms (g -0.08, CI -0.29 to 0.13, 12 studies) | Very few studies specifically targeting negative symptoms  High level of heterogeneity of the original studies.  No conclusions on effects specific for primary or predominant negative symptoms can be drawn | I |
| Velthorst, et al., 2015 [12] | Meta-analysis of randomized controlled trials | P: Patients with schizophrenia spectrum or other non-organic psychotic disorder  I: CBT targeting one of the following outcome domains: psychotic symptomatology, negative symptoms, functioning, self-esteem or cannabis use  C: Treatment as usual, active or inactive control  O: Negative symptoms, reported with PANSS, SANS or BPRS | Primary outcome: No significant effect in studies with negative symptoms as a secondary outcome (Hedges’ g 0.093, CI -0.028 to 0.214; 28 studies, N=1850) and in studies specifically focusing on negative symptoms (g 0.157, CI -0.10 to 0.409; 2 studies, N=238).  Additional outcomes: Meta-regression revealed that stronger treatment effects were associated with earlier year of publication, lower study quality and with CBT provided individually (as compared with group-based) | Very few studies specifically targeting negative symptoms  No conclusions on effects specific for primary or predominant negative symptoms can be drawn  High level of heterogeneity of the original studies | I |
| Lutgens, et al., 2017 [13] | Meta-analysis of randomized controlled trials | P: Patients with schizophrenia spectrum or other non-organic psychotic disorder  I: Investigation of a psychological or psychosocial intervention, as an add-on intervention to antipsychotic treatment (here we report CBT only)  C: Treatment as usual, active or inactive control  O: Report of negative symptoms using any valid scale | Primary outcome: Compared with treatment as usual cognitive-behavioural therapy (SMD -0.34, CI -0.55 to -0.12; 16 studies, N=1588) provide significant benefit.  Additional analyses: A significant benefit for CBT was found in comparison to TAU but not in comparison to an active control. | Very few studies specifically targeting negative symptoms  Not all available studies were included  High level of heterogeneity of the original studies.  No conclusions on effects specific for primary or predominant negative symptoms can be drawn | I |

**eTable 6. Evidence for social skills training.**

| **Study** | **Type of Study** | **Methods** | **Results** | **Limitations** | **Evidence Level** |
| --- | --- | --- | --- | --- | --- |
| Lutgens, et al., 2017 [13] | Meta-analysis of randomized controlled trials | P: Patients with schizophrenia spectrum or other non-organic psychotic disorder  I: Investigation of a psychological or psychosocial intervention, as an add-on intervention to antipsychotic treatment (here we report skills training only)  C: Treatment as usual, active or inactive control  O: Report of negative symptoms using any valid scale | Primary outcome: Compared with treatment as usual skills-based training (SMD -0.44, CI -0.77 to -0.10; 13 studies) provide significant benefit. | Heterogeneous interventions including social skills training, occupational therapy, cognitive adaptation training and vocational training.  High level of heterogeneity of the original studies in other aspects of the design.  Very few studies specifically targeting negative symptoms  Not all available studies were included  No conclusions on effects specific for primary or predominant negative symptoms can be drawn. | I |
| Turner, et al., 2018 [14] | Meta-analysis of randomized controlled trials | P: Patients with psychotic disorders including schizophrenia, schizoaffective disorder, delusional disorder, brief psychotic disorder, or psychosis not otherwise specified.  I: Designated as Social skills training (SST) or other interventions primarily intended to improve social performance  C: Active control, treatment-as-usual (TAU) or wait-list control.  O: Negative symptoms were the primary outcome. | Primary outcome: For negative symptoms SST demonstrated superiority over all comparators pooled (g 0.191, CI 0.043 to 0.338; 17 studies). The effect was significant for comparison with TAU (g 0.3, CI 0.07 to 0.54; 5 studies) and trend-level for comparison with active (g 0.18, CI 0.009 to 0.37; 10 studies).  Additional results:  Restriction to high-quality studies increased the effect size for negative symptoms. | High level of heterogeneity of the original studies.  No conclusions on effects specific for primary or predominant negative symptoms can be drawn.  Comparison between different SST programs limited by the small number of studies available for each.  Many small studies were included, exposing to an inflated effect sizes. | I |

**eTable 7. Evidence for cognitive remediation.**

| **Study** | **Type of Study** | **Methods** | **Results** | **Limitations** | **Evidence Level** |
| --- | --- | --- | --- | --- | --- |
| Cella, et al., 2017 [15] | Pairwise and network meta-analysis of randomized controlled trials | P: Patients with schizophrenia or schizoaffective disorder (>75% of patients)  I: Cognitive remediation (both computerized and pencil/paper)  C: Treatment as usual, active or inactive control  O: Negative symptoms measured with a validated scale | Primary outcome: At post-treatment cognitive remediation favored over TAU / active control conditions for negative symptoms (Hedge’s g 0.35, CI 0.25 to 0.44; p<0.01; studies=45, N=2511); results confirmed after exclusion of two outliers (g 0.30, CI 0.22 to 0.36; 42 studies, N=2318)  At follow-up cognitive remediation favored over TAU / active control conditions (g -0.52, CI 0.23 to 0.80; studies=15, N=808); results confirmed after exclusion of one outlier (Hedge’s g -0.36, CI 0.21 to 0.51; 14 studies, N=737)  Network meta-analysis shows superiority of cognitive remediation over both TAU and active control conditions  High quality studies integrating CR with rehabilitation effects show largest effect size | High level of heterogeneity of the original studies.  None of the studies targeted negative symptoms as primary outcome.  No evidence for patients with primary or persistent negative symptoms. | I |

**eTable 8. Evidence for physical exercise.**

| **Study** | **Type of Study** | **Methods** | **Results** | **Limitations** | **Evidence Level** |
| --- | --- | --- | --- | --- | --- |
| Dauwan, et al., 2016 [16] | Meta-analysis of randomized controlled trials | P: Patients with a diagnosis of schizophrenia or schizophrenia spectrum disorder  I: Any type of exercise as an intervention on psychiatric symptoms  C: Any comparison (e.g. TAU, waitlist, active and inactive control)  O: Total, general, positive and negative symptoms (the latter measured with SANS or PANSS) | Primary outcome: For negative symptom scores exercise was superior to overall controls in improving negative symptoms (g 0.49, IC 0.31 to 0.67; 18 studies, N = 765), active (g 0.33, CI not reported; 10 studies, N=395) and passive (g 0.89, CI not reported; 12 studies, N=459) control conditions. | Original studies with high heterogeneity  Very broad spectrum of exercise interventions  Original studies mostly of limited quality  No information on primary or predominant negative symptoms | I |
| Vogel, et al., 2019 [17] | Meta-analysis of randomized controlled trials | P: Patients with schizophrenia or schizoaffective disorder  I: Physical exercise including mind-body exercise, aerobic exercise and resistance training  C: Treatment as usual, active or inactive control  O: Negative symptoms as measured with the PANSS or the SANS | Primary outcome: For negative symptoms physical exercise favored over TAU (g 0.43, CI 0.2 to 0.67; 22 studies, N=1249).  Additional analyses: Mind-body exercise favored over TAU (g 0.46, CI 0.13 to 0.79; 12 studies).  Aerobic exercise favored over TAU (g 0.34, CI 0.08 to 0.60; 14 studies) | Original studies with high heterogeneity  Very broad spectrum of exercise interventions  Original studies mostly of limited quality  No information on primary or predominant negative symptoms | I |
| Sabé, et al., 2019 [18] | Meta-analysis of randomized controlled trials | P: Patients with schizophrenia or schizoaffective disorder  I: Studies comparing different physical exercise interventions (anaerobic exercise, aerobic exercise, or non-specified exercise) as add-on therapy to ongoing medication  C: Treatment as usual, active or inactive control  O: Effect of adjunctive mind-body therapies on negative symptoms measured with a validated scale | Primary outcome: A beneficial effect of physical exercise on negative symptoms compared to control conditions is found (SMD, -0.24, CI -0.43 to -0.06; 17 studies, N=954). This result is essentially driven by the aerobic subgroup (SMD -0.31, CI -0.54 to -0.09; 12 studies, N=508).  Additional analyses: No specific results was found when considering moderators (duration of practice, mean age, mean baseline negative symptom severity, and change in VO_2_ max). | Original studies with high heterogeneity regarding the type of intervention.  Original studies mostly of limited quality  No information on primary or predominant negative symptoms | I |

**References**

1. Leucht S, Leucht C, Huhn M, Chaimani A, Mavridis D, Helfer B, et al. Sixty Years of Placebo-Controlled Antipsychotic Drug Trials in Acute Schizophrenia: Systematic Review, Bayesian Meta-Analysis, and Meta-Regression of Efficacy Predictors. Am J Psychiatry. 2017;174(10):927-42.

2. Galling B, Roldan A, Hagi K, Rietschel L, Walyzada F, Zheng W, et al. Antipsychotic augmentation vs. monotherapy in schizophrenia: systematic review, meta-analysis and meta-regression analysis. World Psychiatry. 2017;16(1):77-89.

3. Krause M, Zhu Y, Huhn M, Schneider-Thoma J, Bighelli I, Nikolakopoulou A, et al. Antipsychotic drugs for patients with schizophrenia and predominant or prominent negative symptoms: a systematic review and meta-analysis. Eur Arch Psychiatry Clin Neurosci. 2018;268(7):625-39.

4. Huhn M, Nikolakopoulou A, Schneider-Thoma J, Krause M, Samara M, Peter N, et al. Comparative efficacy and tolerability of 32 oral antipsychotics for the acute treatment of adults with multi-episode schizophrenia: a systematic review and network meta-analysis. Lancet (London, England). 2019.

5. Helfer B, Samara MT, Huhn M, Klupp E, Leucht C, Zhu Y, et al. Efficacy and Safety of Antidepressants Added to Antipsychotics for Schizophrenia: A Systematic Review and Meta-Analysis. Am J Psychiatry. 2016;173(9):876-86.

6. Gregory A, Mallikarjun P, Upthegrove R. Treatment of depression in schizophrenia: systematic review and meta-analysis. Br J Psychiatry. 2017;211(4):198-204.

7. Galling B, Vernon JA, Pagsberg AK, Wadhwa A, Grudnikoff E, Seidman AJ, et al. Efficacy and safety of antidepressant augmentation of continued antipsychotic treatment in patients with schizophrenia. Acta Psychiatr Scand. 2018;137(3):187-205.

8. Aleman A, Enriquez-Geppert S, Knegtering H, Dlabac-de Lange JJ. Moderate effects of noninvasive brain stimulation of the frontal cortex for improving negative symptoms in schizophrenia: Meta-analysis of controlled trials. Neurosci Biobehav Rev. 2018;89:111-8.

9. Kennedy NI, Lee WH, Frangou S. Efficacy of non-invasive brain stimulation on the symptom dimensions of schizophrenia: A meta-analysis of randomized controlled trials. Eur Psychiatry. 2018;49:69-77.

10. Osoegawa C, Gomes JS, Grigolon RB, Brietzke E, Gadelha A, Lacerda ALT, et al. Non-invasive brain stimulation for negative symptoms in schizophrenia: An updated systematic review and meta-analysis. Schizophr Res. 2018.

11. Jauhar S, McKenna PJ, Radua J, Fung E, Salvador R, Laws KR. Cognitive-behavioural therapy for the symptoms of schizophrenia: systematic review and meta-analysis with examination of potential bias. Br J Psychiatry. 2014;204(1):20-9.

12. Velthorst E, Koeter M, van der Gaag M, Nieman DH, Fett AK, Smit F, et al. Adapted cognitive-behavioural therapy required for targeting negative symptoms in schizophrenia: meta-analysis and meta-regression. Psychol Med. 2015;45(3):453-65.

13. Lutgens D, Gariepy G, Malla A. Psychological and psychosocial interventions for negative symptoms in psychosis: systematic review and meta-analysis. Br J Psychiatry. 2017;210(5):324-32.

14. Turner DT, McGlanaghy E, Cuijpers P, van der Gaag M, Karyotaki E, MacBeth A. A Meta-Analysis of Social Skills Training and Related Interventions for Psychosis. Schizophr Bull. 2018;44(3):475-91.

15. Cella M, Preti A, Edwards C, Dow T, Wykes T. Cognitive remediation for negative symptoms of schizophrenia: A network meta-analysis. Clin Psychol Rev. 2017;52:43-51.

16. Dauwan M, Begemann MJ, Heringa SM, Sommer IE. Exercise Improves Clinical Symptoms, Quality of Life, Global Functioning, and Depression in Schizophrenia: A Systematic Review and Meta-analysis. Schizophr Bull. 2016;42(3):588-99.

17. Vogel JS, van der Gaag M, Slofstra C, Knegtering H, Bruins J, Castelein S. The effect of mind-body and aerobic exercise on negative symptoms in schizophrenia: A meta-analysis. Psychiatry Res. 2019;279:295-305.

18. Sabe M, Kaiser S, Sentissi O. Physical exercise for negative symptoms of schizophrenia: Systematic review of randomized controlled trials and meta-analysis. Gen Hosp Psychiatry. 2020;62:13-20.
